# Effects of dulaglutide combined with insulin degludec on glucose fluctuations and appetite in type 2 diabetes

**DOI:** 10.3389/fendo.2023.1130470

**Published:** 2023-05-15

**Authors:** Jinxin Huang, Fei Hua, Xiaohong Jiang, Xingguang Zhang, Minxing Yang, Long Wang, Xiaolin Huang, Kaiming Luo

**Affiliations:** ^1^ Endocrinology Department, The Third Affiliated Hospital of Soochow University, Changzhou, China; ^2^ Endocrinology Department, The Seventh Medical Center of PLA General Hospital, Beijing, China

**Keywords:** dulaglutide, persistence with treatment, glucose fluctuation, time in range, appetite, type 2 diabetes mellitus

## Abstract

**Introduction:**

The aim of this study was to describe appetite and glucose fluctuation in type 2 diabetes mellitus patients initiating treatment with dulaglutide combined with insulin degludec.

**Methods:**

This retrospective study of patients identified adults starting treatment with once-weekly (QW) dulaglutide combined with insulin degludec (experimental group) or insulin degludec alone (control group). Patients were followed for up to 6 months from treatment initiation. The clinical characteristics of patients, treatment patterns, CGM data, and appetite scores were obtained for the two groups.

**Results:**

A total of 236 patients were included in this study. SDBG, MAGE, LAGE, and PPGE of the experimental group were lower than the control group’s (P < 0.05). The proportions of patients achieving a time in range (TIR) of ≥70% in the experimental group were higher than in the control group, with 43% and 10% on the second day, 88% and 47% on the fourth day, 95% and 47% on the seventh day, and 100% and 67% on the tenth day, respectively. Significant associations existed between TIR and the prevalence of islet function. At six months, 89.2% of patients in the experimental group were still using dulaglutide. Appetite decreased significantly at 1 week and increased at 3 months after treatment with dulaglutide.

**Conclusion:**

Dulaglutide combined with insulin degludec significantly reduces glucose fluctuations in patients with type 2 diabetes mellitus and improves the TIR rate. However, the treatment on appetite could decrease in the first three months.

## Introduction

Diabetes mellitus is a metabolic disorder with an increasing global prevalence and cause serious health risk. The prevalence of DM in China has increased from 11.6% in 2010, and 12.8% in 2017 for decades (1, 2). The projected number of diabetic population in China would increase rapidly from 141.65 million in 2020 to 202.84 million in 2050 (3). With the growing number of diabetes, the increasing cost of its prevention and control has increased the health burden (2). Therefore, it is very important to control the development of diabetes. As we know, the glycosylated hemoglobin (HbAIc) was established by studies such as the diabetes control and complications trial (DCCT) and British prospective diabetes study (UKPDS) as predictors of diabetic chronic complications. However, the same HbAlc level did not reflect the same risk of complications (4).

Recent studies have shown that increased blood glucose fluctuation is an important risk factor for hyperglycemia and adverse outcome in diabetic patients (5–7) and its harm is independent of glycosylated hemoglobin. There are many factors affecting glucose fluctuation, among which postprandial hyperglycemia and hypoglycemia are two important causes of glucose fluctuation in diabetic patients (8). High postprandial blood glucose, poor islet function, use of insulin/insulin analogues or insulin secretagogue, high risk of hypoglycemia, long course of disease, and advanced age diabetes patients generally have high blood glucose fluctuation (9).Current studies have shown that combined with α-glucosidase inhibitors (acarbose) on the basis of sulfonylurea secretrogues, hypoglycemia can be reduced and glucose fluctuation can be significantly improved. Patients treated with insulin/insulin analogues are significantly improved by combination with alpha-glucosidase inhibitors (acarbose)(10). As a new ultra-long-acting insulin analog with a half-life of 25 h, insulin degludec itself also has a stable pharmacokinetic curve, which can effectively reduce hypoglycemic events and maintain glucose homeostasis (11). However, But the risk of hypoglycemia with insulin still worsens glucose fluctuations.

Dulaglutide is a novel long-acting GLP-1 receptor agonist, which has the function of glucose-dependent promotion of insulin secretion, and can reduce the risk of hypoglycemia compared with insulin therapy. AWARD series studies (12–16) have confirmed that weekly subcutaneous injection of dulaglutide has a good effect on decreasing glucose levels. One week of GLP-1 use can influence appetite and reduce food preference and body weight in individuals with obesity (17), however, the studies on the effect of dulaglutide combined with insulin degludec on glucose fluctuations and appetite in the Chinese population are scarce.

The aim of our study was to explore the impact of glucose fluctuation and appetite on the use of dulaglutide combined with insulin degludec in T2DM patients. Flash glucose monitoring (FGM) was used to observe glucose fluctuations and the time in range (TIR) in included patients, while, visual analogue scale (VAS) (17) was used to evaluate the changes in patients’ appetite.

## Patients and methods

### Patients

In this research, patients who were diagnosed with type 2 diabetes and required insulin therapy to control hyperglycemia were included. All patients were selected from the clinical endocrinology center of our hospital from January 2020 to June 2021 in the Outpatient Department of Endocrinology.

Detailed inclusion criteria were male or female patient age 18-75 years; All subjects were treated with insulin (Degludec insulin injection) in combination with Oral antidiabetes drugs (HbA1c > 7.0%). On this basis, 112 patients were additionally given 1.5mg dulaglutide skin injection once a week. Diabetes met the 1999 World Health Organization (WHO) diagnostic criteria; Patients were excluded if acute complications of diabetes or other stressful conditions; severe liver and kidney function damage; cardiovascular disease; cerebrovascular disease; a serum calcitonin of 50 pg/ml on admission; a history of pancreatitis; a triglyceride (TG) level of 5.6 mmol/l or greater; a family history of medullary thyroid carcinoma or multiple endocrine neoplasia syndrome (MEN2); a history of diabetic gastroparesis or inflammatory bowel disease; comorbid conditions, such as a gastrointestinal or mental disease or a malignant tumor; pregnancy or planned pregnancy; breastfeeding; and immune deficiency. This study is consistent with the principles of the Declaration of Helsinki Amendments and were approved by the independent ethics committee of the Third Affiliated Hospital of Soochow University and institutional review board of the study center (Approval number was: 2018-007). Written informed consent was obtained from each patient.

### Study design

The included patients were treated with insulin degludec (control group) or insulin degludec combined with dulaglutide (experimental group) to achieve HbA1c < 7% without hypoglycemia. All patients adjusted the dose of insulin degu within the first week of inclusion and patients in the experimental group received 1.5 mg dulaglutide once weekly (QW) to achieve the expected FPG target. The dosage of dulaglutide was maintained for six months after one week of use without nausea, abdominal pain, or other gastrointestinal reactions. During the treatment period, the dose of insulin degludec was adjusted to achieve the glucose target stably. Patients were followed up at two weeks, one month, three months, and six months after treatment. In this study, the long-term evaluated efficacy indicators including the percentage of patients receiving degludec therapy, body mass index, appetite changing scale, islet function. Moreover, short-term glucose fluctuations and the time in range (TIR) after the first 2 weeks treatment were evaluated. The glucose control target was an FPG of 4.4-6.1 mmol/l, with a 2hPG <10.0 mmol/l. Patient was considered to achieve glucose control when FBG was ≥7.0 mmol/l or <4.4 mmol/l and non FBG was >10 mmol/l; glucose was considered to have not reached the standard when FBG was 4.4-7.0 mmol/l and non FBG was ≤10.0 mmol/l.

### Insulin dose titration

The initial administered total dose of insulin degludec was 0.2-0.3 U/kg. The dose was adjusted according to the fasting glucose level of the patients. When the FBG level was < 6.1 mmol/l, the insulin dosage was gradually decreased (2-4 U) or insulin was stopped, and the stopping time and follow-up medication status were recorded.

### Glucose profile monitoring using CGM

Continuous glucose monitoring system (CGMS) was applied on all the subjects for 14 days. From the second day of admission, finger-stick glucose testing was performed seven times a day, and the insulin dose was adjusted according to the finger-stick-measured glucose levels. At the same time, patients were required to wear a flash glucose monitor for 14 days. HbA1c and finger-stick-measured glucose were monitored, and the time of glucose reaching the target range (i.e., the TIR) was calculated. The mean amplitude of glycemic excursion (MAGE), the standard deviation of blood glucose (SDBG), the largest amplitude of glycemic excursion (LAGE), and postprandial glucose excursion (PPGE), and absolute means of daily differences (MODD) were calculated by a CGMS. The TIR standard used in this study was 3.9-10 mmol/l.

### Demographic data

Demographic and general clinical data such as the gender, age, body mass index (BMI), weight, systolic pressure (SBP), diastolic pressure(DBP),low density lipoprotein cholesterol (LDL-c), triglyceride (TG), total cholesterol (TC), Apolipoprotein A1,apolipoprotein B, the fasting C-peptide and 2-h C-peptide (2hC-P) (radiometric monitoring, the continuous glucose monitoring system (CGMS) of MiniMed company) subjects were recorded. Fasting plasma glucose (FPG) and 2-h postprandial glucose (2hPG) were monitored daily, and the insulin dosage was adjusted according to these glucose parameters. The day following dulaglutide injection, venous blood was collected. Urinary microalbumin was measured by Beckman Coulter Immage automatic immunoanalyzer (rate scattering turbidimetric method), and the mean values of three times at different times were obtained. Urinary protein excretion rate was calculated according to urinary creatinine.

### Appetite assessment

In this study, a visual analogue scale (VAS)(17) was used to evaluate the changes in patients’ appetite. Before and 2 h after a standardized breakfast, appetite parameters (hunger, fullness, satiety, and expected food consumption) were subjectively scored on a 100 mm VAS, with the end of each VAS line representing the most extreme sensation experienced by the patient. Overall appetite suppression scores were calculated based on the four appetite parameters (Appendix S1). VAS scores were repeatedly evaluated on days 2, 4, and 8 and 1, 3, and 6 months after the initiation of dulaglutide treatment.

### Statistical analysis

SPSS 20.0 software was used for statistical analysis. The Kolmogorov–Smirnov test was used to determine whether the measured data followed a normal distribution; If the data were normal distribution data, t-test was used for comparison between two groups. If the data were non-normal distribution data, rank sum test was used for comparison between two groups. The chi-square test or Fisher’s exact probability test was used to compare the counting data. A paired t-test was used for comparisons before and after treatment. Chi-square test was used for qualitative data testing; with all the variables investigated (continuous variables and the binary classification variables) as a relevant variable, TIR as the dependent variable for Logistic regression to analyze the influence of different treatment regimens on glucose fluctuation. P<0.05 was considered statistically significant.

## Results

A total of 262 patients were included for analysis. Among them, 26 participants who discontinued the study, 5 participants withdrew for personal reasons, and 21 participants missed follow-up or lost the data. The other 236 T2DM patients, 128 males, and 108 females, There were 112 patients were assigned to the experimental group and 124 patients assigned to the control group. The demographic comparison showed there was no significant difference in baseline data between the two groups (**Table 1**). They had higher propensity to receive renin-angiotensin-aldosterone system inhibitors, and calcium-channel blockers. The details of oral antidiabetes drug use are presented in **Table 1**. After three months of treatment, the fasting blood glucose were lower in the experimental group than in the control group (7.45 ± 2.26 vs 8.22 ± 2.11mmol/l, respectively).The renal urinary protein excretion rate(UACR) and BMI in the experimental group was significantly reduced compared with the control group, while the fasting C-peptide (FC-P) and postprandial 2hC peptide(2h C-P) levels of the patients in the experimental group were significantly higher than those in the control group (**Supplementary Table 1**).

**Table 1:** Comparison of baseline data between the two groups.

Data	Experimental group	Control group	P-value
Sex (number)	112	124	–
male/female	64/48	64/60	0.434
Age(years)	61.32±12.81	61.62±11.73	0.928
Diabetes durations(years)	9.43±7.21	9.31±7.15	0.872
Current smoker (%)	26.2	27.6	0.874
Use antidiabetes agents(%)			
Oral antidiabetes drugs	57.2	56.4	0.887
Use antihypertension agents(%)			
RAAS inhibitors	44.2	42.8	0.887
Cancium-channel blockers	19.7	20.6	0.861
β-blockers	7.6	8.5	0.816
Diuretics	3.9	3.8	0.710
Use lipid-lowering agents(%)			
Statins	24.8	24.5	0.968
Fibrates	11	10	0.818
BMI (kg/m^2^)	26.54±3.35	26.50±3.01	0.124
Weight(kg)	76.14±8.67	77.21±8.13	0.333
TC (mmol/l)	5.07±1.43	4.70±1.34	0.454
TGs (mmol/l)	2.11±0.23	2.72±0.76	0.321
LDL-C (mmol/l)	3.03±0.82	2.83±0.91	0.575
Apolipoprotein A1 (mmol/l)	1.19±0.27	1.24±0.31	0.592
Apolipoprotein B (mmol/l)	1.00±0.28	1.02±0.27	0.864
SBP (mmHg)	135±5	130±3	0.373
DBP (mmHg)	86±2	85±1	0.951
HbA_1_C (%)	10.42±1.32	10.28±1.01	0.282
FBG (mmol/l)	10.36±2.74	10.81±5.53	0.147
Creatinine (µmol/l)	78.87±6.12	81.21±11.65	0.723

BMI, Body mass index; TC, Total cholesterol; TGs, Triglycerides; LDL-C, Low-density lipoprotein; SBP, Systolic blood pressure; DBP, Diastolic blood pressure; HbA_1_C, Haemoglobin A1c; FBG, Fasting blood glucose.

### Glucose comparison after two weeks treatment

**Table 2** shows the detailed data of all of the GV measures including largest amplitude of glycemic excursion (LAGE), SDBG, and MAGE differed significantly across the various groups(all P for trend,0.05). At the beginning of treatment, compared with the control group, the experimental group had lower LAGE(8.79 ± 1.74 vs 9.87 ± 3.17mmol/l),MAGE(6.22 ± 1.82 vs 11.6 ± 3.33mmol/l), SDBG(3.71 ± 0.96 vs 5.41 ± 0.84mmol/l), PPGE(4.60 ± 2.89 vs 6.85 ± 1.99mmol/l),while, at 2 weeks(day11-13), compared with the control group, the experimental group also had lower LAGE(6.52 ± 0.85 vs 8.17 ± 1.98mmol/l),MAGE(4.05 ± 1.04 vs 5.19 ± 0.98mmol/l),SDBG (2.68 ± 0.73 vs 3.27 ± 0.62mmol/l),PPGE(2.33 ± 0.51 vs 4.25 ± 1.33mmol/l). At the 2 weeks(day11-13), LAGE,SD,MAGE in both groups decreased rapidly than day 2-4. But the absolute means of daily differences(MODD) had no significant difference between the two groups.

**Table 2:** Comparison of glucose levels and their fluctuation indices between the two groups at 2 weeks ($\bar{\text{x}}$ ± s): Data from FGM.

Group	Day 2-4 (mmol/l)					Day 11-13 (mmol/l)				
	LAGE	MAGE	SDBG	PPGE	MODD	LAGE	MAGE	SDBG	PPGE	MODD
Experimental	8.79 ± 1.74	6.22 ± 1.82	3.71 ± 0.96	4.60 ± 2.89	2.08 ± 1.20	6.52 ± 0.85	4.05 ± 1.04	2.68 ± 0.73	2.33 ± 0.51	2.21 ± 1.45
Control	9.87 ± 3.17	11.6 ± 3.33	5.41 ± 0.84	6.85 ± 1.99	1.78 ± 0.35	8.17 ± 1.98	5.19 ± 0.98	3.27 ± 0.62	4.25 ± 1.33	2.03 ± 1.21
t	-1.64	-5.60	-3.97	-2.869	0.606	-1.85	-2.25	-1.665	-2.287	0.627
P	0.048	0.000	0.000	0.040	0.549	0.045	0.031	0.1	0.007	0.572

FGM, Flash glucose monitoring; LAGE, largest amplitude of glycemic excursion; MAGE, mean amplitude of glycemic excursion; SDBG, standard deviation of blood glucose; PPGE, Postprandial glucose excursion; MODD, Absolute means of daily differences.

### TIR, TAB and TBR at different time within two weeks

Glucose data on the 2nd, fourth, seventh, and 10th days after continuous glucose monitoring were collected and analyzed. The proportion of patients reaching the glucose standard(TIR≥70%)increased daily during the treatment period in the two groups (**Table 3**). With the extension of treatment time, the trend in patient compliance became more obvious. The proportion of patients with a TIR≥70% in the experimental group and the control group was 43% and 10% on the second day, 88% and 47% on the fourth day, 95% and 47% on the seventh day, and 100% and 67% on the tenth day, respectively. The experimental group had lower TBR proportion than the control group and lower TAR proportion on the 7th and 10th days (**Table 3**). The compliance rate and the proportion of patients reaching the 2hPG standard in the experimental group were significantly higher than those in the control group probably because of good glucose control. The proportions of patients in the experimental and control groups reaching the 2hPG standard at 4, 7, 10, and 13 days were 44% and 32%, 65% and 43%, 86% and 63%, and 100% and 68%, respectively. (**Tables 3**). In a multinomial logistic regression model with patients without use of GLP-1 as the reference group, significant associations existed between TIR and the prevalence of islet function (baseline: P = 0.019;3months: P = 0.017; 6 months:P=0.001) after adjusting for age, sex, BMI, diabetes duration, HbA1c, blood pressure, and lipid profile (**Table 4**).

**Table 3:** Comparison of the ratio of each index in the two groups at different times within 2 weeks (%).

Group	Number	TIR≥70%						TBR						TAR					
		Day 2	Day 4	Day 7		Day 10		Day 2	Day 4		Day 7	Day 10		Day 2		Day 4	Day 7	Day 10	
Experimental	112	43	88	95		100		24	10		13	10		95		88	64	44	
Control	124	10	47	47		67		38	29		38	38		81		89	80	60	
		2hPG ≤ 10mmol/l																
		Day2			Day4					Day7					Day10			
	112	44			65					86					100			
	124	32			43					63					68			

TIR, Time in range (3.9-10 mmol/l) < TIR, glucose<3.9mmol/l, >TIR, glucose>10mmol/l.

**Table 4:** Associations between TIR and various stage of islet function(the fasting C-peptide level) after controlling for confounding factors.

		Baseline(FC-P)		3months(FC-P)	6 months(FC-P)	
	OR(95%CI)	P-value	OR(95%CI)	P-value	OR(95%CI)	P-value
Model1						
TIR*	0.94(0.88-0.99)	0.019	0.90(0.83-0.99)	0.017	0.91(0.87-0.97)	0.001
Model2						
TIR*	0.95(0.89-1.02)	0.045	1.32(1.02-1.61)	0.032	0.98(0.84-1.23)	0.006
SD	0.56(0.33-0.93)	0.028	0.53(0.2-0.94)	0.023	0.52(0.35-0.84)	0.004
Model3						
TIR*	0.93(0.82-1.01)	0.037	0.81(0.647-0.990)	0.03	0.93(0.88-0.97)	<0.001
MAGE	0.55(0.33-0.92)	0.016	0.56(0.29-0.96)	0.032	0.63(0.50-0.95)	0.006

Model 1 was adjusted for age, sex, BMI, diabetes duration, blood pressure, lipid profile, and HbA1c.Model2 includes all variables in model1 plus SD. Model 3 includes all variables in

Model1 plus MAGE.*ORs and P values were estimated in TIR(0-100%)

### Use of insulin degludec

For patients achieved glucose control, the proportion of patients using insulin degludec decreased in both groups, but the decrease was more evident in the experimental group (experimental group: 84.8%, 70.5%, 34.8%, and 7.14% at the second week, first month, third month and sixth month; control group: 100%, 74.2%, 53.5%, and 31.4% respectively) (**Table 5**).

**Table 5:** Percentage of patients using insulin degludec when glucose reached the standard in the two groups (%).

Group	Number	Day 14	Day 30	Day 90	Day 180
Experimental	112	84.8	70.5	34.8	7.14
Control	124	100	74.2	53.2	31.14
P-value	–	0.000	0.558	0.010	0.000

### Appetite change

**Figure 1:**
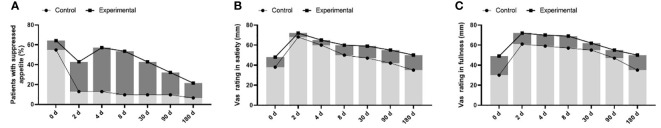
comparison of appetite changes between control group (insulin degludec alone) and experimental group (once weekly dulaglutide combined with insulin degludec). **(A)** the suppressed appetite was significantly higher in the experimental group (55% and 12%). **(B, C)** Patients’ fullness and satiety VAS scores in the experimental group were significantly higher than those in the control group.

**Figure 2:**
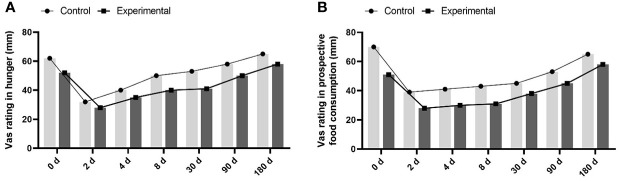
the hunger score and the expected food consumption score between the 2 groups. **(A)** increased hunger score in experimental group compared to control group. **(B)** increased expected food consumption score in experimental group compared to control group.

The proportion of patients with suppressed appetite in the experimental group was significantly higher than that in the control group (55% and 12%, respectively) within one week (**Figure 1A**). Patients’ fullness and satiety VAS scores in the experimental group were significantly higher than those in the control group from the second day of treatment (**Figures 1B, C**). In contrast, the hunger score increased, and the expected food consumption score increased (**Figures 2A, B**). However, until day 90, compared with the control group, eating desire and hunger scores increased, while satisfaction and satiety decreased in the experimental group, suggesting that appetite recovered at approximately 90 days.

## Discussions

Glucagon-like peptide (GLP)-1 receptor agonists have been one of the most popular hypoglycemic agents in recent years (18). The structure of dulaglutide is such that glycine replaces arginine residues at the 36th position of natural GLP-1, preventing it from being decomposed by the DPP-4 enzyme and prolonging the activity of incretin. The delay of gastric emptying and central appetite suppression can help patients with type 2 diabetes mellitus (T2DM) achieve better glycemic control (19). The AWARD series of studies confirmed that patients receiving a single injection of dulaglutide or dulaglutide combined with glucose control drugs such as metformin alone, metformin combined with pioglitazone, insulin alone, or the multiple daily injection (MDI) regimen (12, 13, 20) showed an effective decrease in the average glucose and HbA1c levels.

In clinical practice, Proper use of insulin is crucial to the influence of glucose fluctuations. Some patients with T2DM have been treated with long-acting insulin combined with multiple Oral antidiabetes drugs, but their glucose is still not well controlled. Increasing the dose of insulin or Oral antidiabetes drugs may lead to further weight gain or increase the risk of hypoglycemia, causing poor glucose fluctuation. Insulin degludec, as a new ultra-*long* insulin analogue, compared with other long-acting insulin, such as insulin glargine or neutral protamine Hagedorn insulin, can induces fewer hypoglycemic events and can better maintain glucose homeostasis. In our study, the glucose standard deviation, MAGE, maximum glucose fluctuation range, and postprandial glucose fluctuation range were significantly decreased in patients treated with insulin degludec combined with dulaglutide. However, there was no significant difference in the fluctuation range of daytime glucose(MODD), which may be related to the use of insulin degludec in both groups. It should be noted that the significant decrease in glucose fluctuation in patients with the addition of dulaglutide was mainly related to MAGE and PPGE, suggesting that the addition of dulaglutide to make TIR reach the standard, and its contribution to glucose fluctuation may be reflected in the control of postprandial glucose levels and fluctuations.

A series of methods can effectively reduce glucose fluctuation in diabetic patients. Shi, F. H (21) found that the clinical pharmacist intervention contributed to improved outcomes, specifically, in reducing blood glucose fluctuations and potential hypoglycemia risk. However, some drugs have advantages in controlling glucose homeostasis. Acarbose and siglitine were reported that can reduce glucose fluctuation and improve islet β-cell function (22). Nauck, M (14) found that after the addition of sitagliptin to the treatment regimen of patients with diabetes with poor glucose control on oral antidiabetes drugs, it was found that the standard deviation of 24-h glucose, the 24-h mean glucose fluctuation range, and the glucose fluctuation range of patients decreased significantly.GLP-1 signaling in the central nervous system can control glucose uptake and production in peripheral tissues, and dulaglutide also plays a role through GLP-1 receptor signaling pathway (23), which may be similar to DPP4 inhibitors in keeping glucose stable. Recent studies suggest that GLP-1 acting can directly effect the brain’s feeding center, affecting the perception of the reward value of food (24). The activation of GLP-1 receptors in the human brain helps to regulate appetite and food reward (25, 26). Our study found that appetite was significantly inhibited within one week, patient satiety and satisfaction were enhanced, in the patients treated with dulaglutide combined with insulin degludec, which may be related to delayed food absorption. Patients in the experimental group recovered their appetite inhibition around the third month. This may also be one of the important reasons why dulaglutide can reduce glucose fluctuation especially postprandial glucose excursion.

Some natural compounds may be beneficial to glucose fluctuations, and active ingredients previously found in Chinese herbal medicines can significantly reduce glucose and urinary protein levels in patients and animal models (27, 28), such as, a coumarin from hydrangea paniculata, slows down the progression of urinary protein exudation by anti-inflammatory effects and inhibiting immune complex deposition. Our study also found that urinary protein excretion rate in the experimental group was significantly reduced compared with the control group, suggesting that dulaglutide is beneficial to improve renal urinary protein exudation in diabetic patients, which was consistent with previous studies (29, von 30). In addition, we also found that the FC-P and 2hC-P levels of the patients in the experimental group were significantly higher than those in the control group after 3 and 6 months of treatment. The HbA1c was reported closely related to the MAGE and islet function in patients with T2DM (31). Our study found that TIR was significantly related to the islet function adjusting for age, sex, BMI, diabetes duration, blood pressure, lipid profile, and HbA1c. when adjusting all variables plus SD and MAGE, the difference was still statistically significant, suggesting that dulaglutide combined with degludec therapy is beneficial to improving the pancreatic function of the patients, which may be related to the significant decrease of glucose fluctuation, the alleviation of hyperglycemic toxicity, and the decrease of oxidative stress and inflammation in the body.

A real-world study in Spain (32) showed that a higher proportion of patients on dulaglutide for hypoglycemic treatment continued to use dulaglutide at 18 months than those on other GLP-1 receptor agonists, such as liraglutide and exenatide, with a more significant reduction in HbA1c and less overall treatment cost. A study in France (Zimner 33) observed the use of different types of GLP-1 receptor agonists in patients and found that the time of continuous administration of dulaglutide was significantly higher than that of liraglutide and exenatide; the median time was 373 days, and the changes in dulaglutide dose were even less when the treatment regimen was changed. Our study also found that in the experimental group, only 7.14% of the patients were being treated with insulin degludec at the six-month follow-up, and 89.2% of the patients continued to be treated with dulaglutide, of whom 27.7% only needed to be treated with dulaglutide once a week, which was similar to studies from other countries (34).

FGM can reflect the glucose level of tissue fluid for up to 14 days in real time and is a new clinical glucose monitoring method similar to continuous glucose monitoring, with similar accuracy (35). The simple application of HbA1c levels as recommended in clinical guidelines is sometimes misleading, but the continuous glucose spectrum can more accurately and intuitively reflect short-term daily glucose fluctuations (36). By extracting FGM data and calculating relevant indices reflecting glucose fluctuations, our study provides new ideas for further in-depth mining of clinical information and evaluation of factors underlying fluctuating data from glucose monitoring and more appropriate and ideal clinical treatment strategies.

However, there are several limitations in our study. Firstly, the study population is not large enough, then more subjects are needed for further study; secondly, our study did not fully reflect the association of all indicators with long-term changes in glucose fluctuations. The possible reasons include the short time of marketing and clinical use of dulaglutide in China and many factors for patients’ acceptance of dulaglutide treatment. Larger, population-based prospective studies are needed in the future to further clarify the effects of dulaglutide combined with insulin degludec on glucose management in China.

## Conclusion

In conclusion, dulaglutide combined with insulin degludec can significantly reduce overall glucose fluctuations and appetite early in diabetes patients, resulting in a higher TIR. Therefore, when glucose of patients with type 2 diabetes is high, we can choose dulaglutide combined with insulin degludec to achieve a more stable hypoglycemic effect.

## Data availability statement

The original contributions presented in the study are included in the article/**Supplementary Materials**. Further inquiries can be directed to the corresponding author.

## Ethics statement

The studies involving human participants were reviewed and approved by ethics committee of the Third Affiliated Hospital of Soochow University. The patients/participants provided their written informed consent to participate in this study. Written informed consent was obtained from the individual(s), and minor(s)’ legal guardian/next of kin, for the publication of any potentially identifiable images or data included in this article.

## Author contributions

FH, XJ conceived and designed the experiments and LW, MY, XZ and JH conducted data collection and JH, XH, KL analyzed the data. The manuscript was written by JH. All authors contributed to the article and approved the submitted version.
