# Supplementary material for: Effects of dulaglutide combined with insulin degludec on glucose fluctuations and appetite in type 2 diabetes
Source: Front Endocrinol (Lausanne). 2023 May 15;14:1130470. doi: 10.3389/fendo.2023.1130470 (PMC10225703; doi:10.3389/fendo.2023.1130470)
Supplement: Supplementary file 1 [file Table_1.docx]

**Supplementary Table 1**. Comparison of clinical date between two groups before and after treatment（3months）

| Group | Time | BMI  (kg/m^2^) | Ccr  (umol/l) | UACR  (mg/g) | FBG  (mmol/l) | FC-P  (ng/ml) | 2hC-P  （ng/ml） |
| --- | --- | --- | --- | --- | --- | --- | --- |
| Experimental | Baseline | 26.54±3.35 | 78.87±6.12 | 73.46(23.57-119.95) | 10.36±2.74 | 1.51±0.63 | 2.84±1.05 |
|  | Change at 3months | 24.12±3.07^*^ | 76.53±6.13 | 53.65(19.61-103.82) ^*#^ | 7.45±2.26^*#^ | 2.78±1.12^*#^ | 3.63±2.17^*#^ |
| Control | Baseline | 26.50±3.01 | 81.21±11.65 | 71.32(25.98-121.45) | 10.81±5.53 | 1.54±0.82 | 2.76±1.24 |
|  | Change at 3months | 26.12±3.21^*#^ | 78.25±7.21 | 69.72(20.92-110.4) | 8.22±2.11^*^ | 2.21±0.87^*^ | 3.31±2.12^*^ |

vs Pre -treatment，^*^P＜0.05;Experimental vs control, ^#^P＜0.05.UACR: Urinary protein excretion rate, FC-P: the fasting C-peptide, 2h C-P: postprandial 2hC peptide
